# Birth-Rate Factors

**Published:** 1921-01-15

**Authors:** B. Dunlop

**Affiliations:** 96 Victoria Street, S.W. 1


*To the Editor of* THE HOSPITAL.

### Birth-rate Factors.

*To the Editor of* THE HOSPITAL.

SIR,—The Registrar-General, in his 1919 Report for England and Wales, suggests that if half a million more children had been born during the war, the population would now be greater by about that amount. How can this be reconciled with the serious food shortage during the war? If there was really a food shortage, a higher birth-rate must have meant a correspondingly higher death-rate. Your comments on the report call for another criticism. Although it may be most desirable to make up our population losses in the war as quickly as possible, times are very hard, as they always are after a great war, and "birth control" is even more urgently needed than usual. It does not help population to have more children than one can provide for.—Yours respectfully, B. DUNLOP, M.B.

96 Victoria Street, S.W. 1.

